# Supplementary material for: Sequential Endoluminal Doxorubicin and Gemcitabine Alternating Weekly with Sequential Mitomycin and Docetaxel for Recurrent Non-Muscle Invasive Urothelial Carcinoma
Source: Cancers (Basel). 2024 Dec 10;16(24):4126. doi: 10.3390/cancers16244126 (PMC11674833; doi:10.3390/cancers16244126)
Supplement: Supplementary file 1 [file cancers-16-04126-s001.zip › cancers-3277043-supplementary.pdf]

Supplementary Table S1. Clinical and pathological features of patients receiving cystectomy and nephroureterectomy.

Specimen Type	Presenting Pathology	Prior Prostatic UC	Post-Surgical Pathology	Prostatic UC at cystectomy	Surgery Reason
Radical Cystectomy	T1HG + CIS	0	T0N0	0	End-stage bladder
Radical Cystectomy	CIS	0	TisN2	0	MIBC
Radical Cystectomy	CIS	0	TisN0	1	Recurrent NMIBC + end-stage bladder
Radical Cystectomy	CIS	1	T4aN0R1	1	Recurrent NMIBC
Radical Cystectomy	HG cytology	0	T2N1	1	Recurrent NMIBC
Radical Cystectomy	HG cytology	0	T0N0	0	End-stage bladder
Partial Cystectomy	CIS	0	T3aN3	0	MIBC
Nephroureterectomy	HG cytology	-	T0N0	-	Persistently abnormal cytology

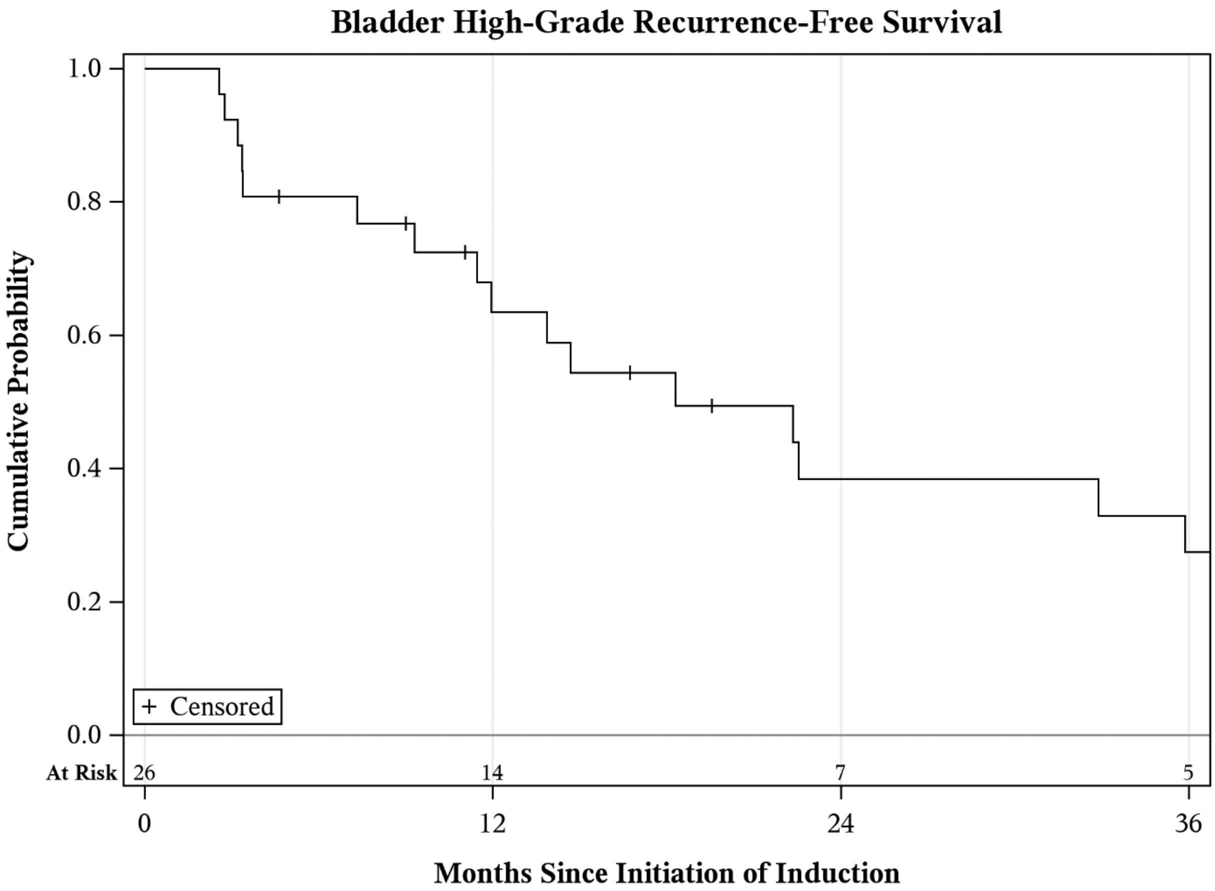

12 Months	24 Months	36 Months
63% (41-79%)	38% (19-58%)	27% (11-48%)

Supplemental Figure S1. High-grade recurrence-free survival following Quad Chemo among patients presenting with disease of the bladder.

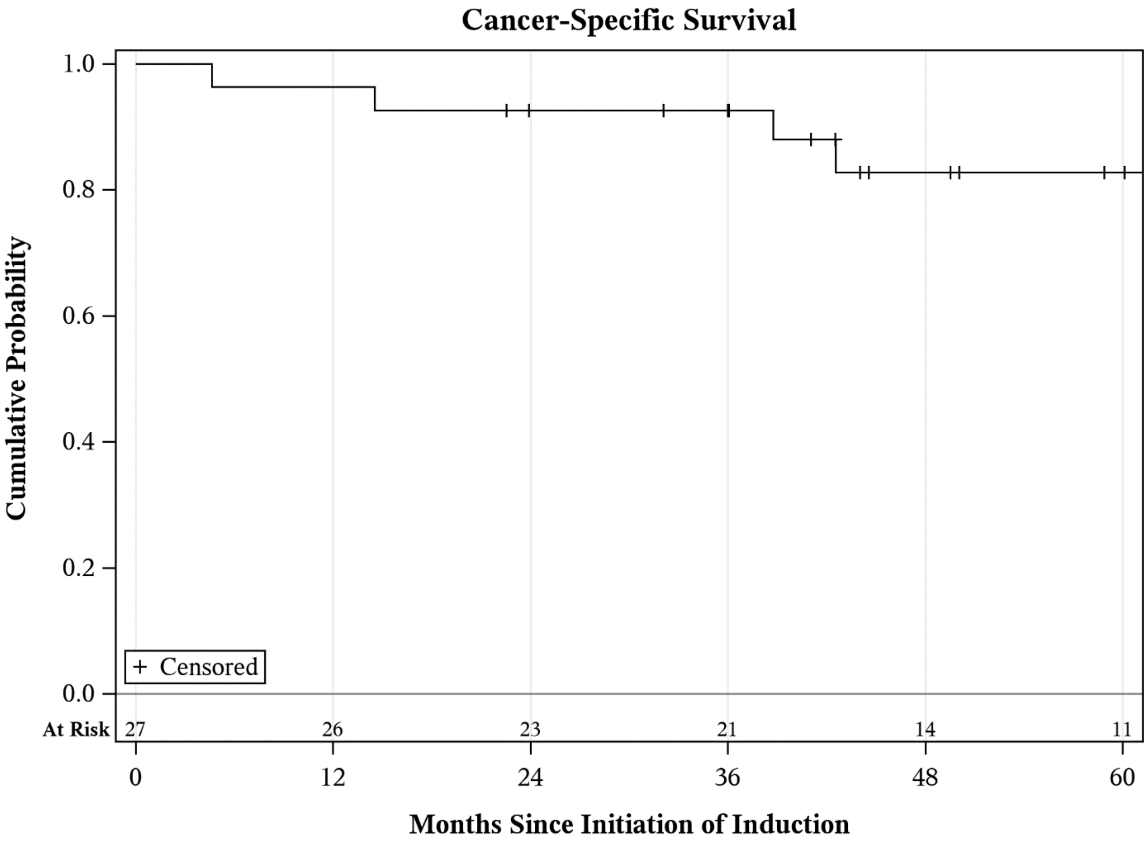

12 Months	24 Months	36 Months	48 Months	60 Months
96% (76-99%)	93% (74-98%)	93% (74-98%)	83% (60-93%)	83% (60-93%)

Supplemental Figure S2. Cancer-specific survival following Quad Chemo treatment.

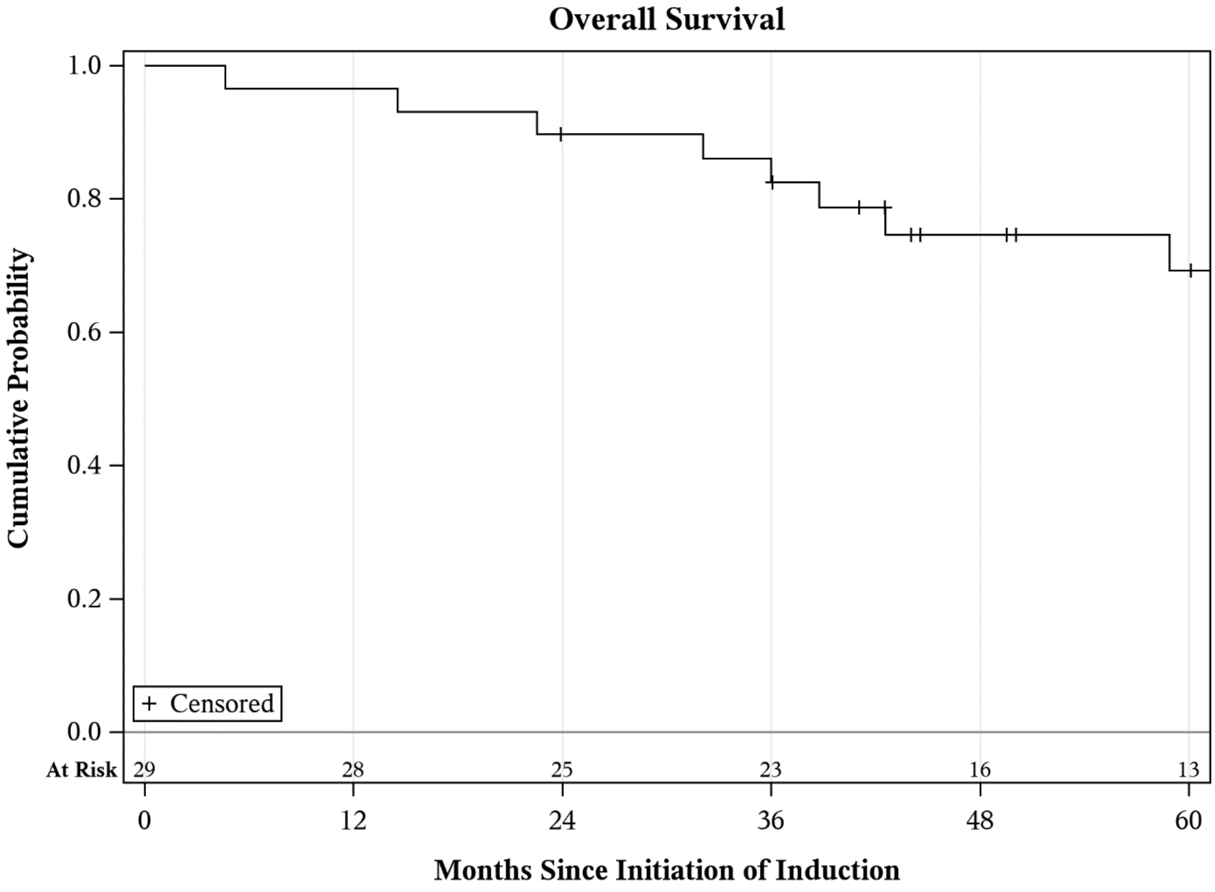

12 Months	24 Months	36 Months	48 Months	60 Months
97% (78-100%)	90% (71-97%)	82% (63-92%)	75% (54-87%)	69% (47-84%)

Supplemental Figure S3. Overall survival following Quad Chemo treatment.
